# Epidemiological monitoring of leprosy indicators in Sergipe (2001–2015): segmented regression analysis^☆^^☆☆^

**DOI:** 10.1016/j.abd.2019.07.015

**Published:** 2020-05-19

**Authors:** Carlos Dornels Freire de Souza, Thiago Cavalcanti Leal, João Paulo Silva de Paiva, Victor Santana Santos

**Affiliations:** a Department of Medicine, Center for the Study of Social and Preventative Medicine, Universidade Federal de Alagoas, Arapiraca, AL, Brazil; b Center for Epidemiology and Public Health, Universidade Federal de Alagoas, Arapiraca, AL, Brazil

**Keywords:** Epidemiology, Leprosy, Time series studies

## Abstract

This study analyzed the trend of leprosy indicators in Sergipe, between 2001 and 2015. It was a time series study that analyzed the trend for general detection coefficient, children under 15 years of age, and new cases with grade 2 disability. The joinpoint model was used. Two (2.6%) municipalities had an increasing trend in general detection coefficient, five (6.6%) had an increasing trend in detection rate in children under 15, and 19 (25.3%) had an increasing trend in detection coefficient of new leprosy cases with grade 2 disability. The findings suggest maintenance of the chain of transmission.

Leprosy is an infectious disease caused by *Mycobacterium leprae*,^1^ which affects the skin and peripheral nerves, resulting in neuro-dermatological lesions and physical disabilities.^2^

Only Brazil has not reached the goal of eliminating leprosy as a public health problem.^3^ In 2017, 26,875 new leprosy cases were diagnosed in Brazil (12.94/100,000 population). Of these, 1718 were diagnosed in children under 15 years of age (3.72/100,000). The detection coefficient of new leprosy cases with grade 2 disability was 9.39/100,000.^4^

In 2017, Sergipe recorded a general detection coefficient of 15.78/100,000 population, a detection coefficient in children under 15 of 2.75/100,000, and detection coefficient of new leprosy cases with grade 2 disability of 16.61/100,000.^4^ The monitoring of these indicators is recommended by the World Health Organization (WHO),^5^ because of the commitments to eliminate the disease signed by Brazil.

This study aimed to analyze the trend of leprosy indicators in the state of Sergipe from 2001 to 2015.

For this purpose, an ecological time series study was performed, with the municipalities of Sergipe (*n* = 75) being the units for analysis. Data were obtained from the National Information System for Notifiable Diseases (SINAN). Three indicators were analyzed: general detection coefficient rate per 100,000 population; detection coefficient rate in children under 15 per 100,000 population, and detection coefficient of new leprosy cases with grade 2 disability per 100,000 population (Table 1). For the temporal analysis, the joinpoint regression model was used. The annual percent change (APC) and the average annual percent change (AAPC) were calculated. A 95% confidence interval (95% CI) and an alpha of 5% were considered. Because of the use of secondary data, the approval of the Research Ethics Committee was not needed.

**Table 1:** Epidemiological indicators and methods selected for the study.

Indicator	Utility	Parameters
New leprosy case detection rate in the general population/100,000 inhabitants.	Measures the strength of the disease's morbidity, magnitude, and trend.	Hyperendemic: ≥40.0/100,000 inhab.
		Very high: 20.00–39.99/100,000 inhab.
		High: 10.00–19.99/100,000 inhab. Medium: 2.00–9.99/100,000 inhab.
		Low: <2.00/100,000 inhab.
		Very high: 20.00–39.99/100,000 inhab.

New leprosy case detection rate in the population under 15 years/100,000 inhabitants.	Measures the strength of recent transmission of the disease and its trend.	Hyperendemic: ≥10.00/100,000 inhab.
		Very high: 5.00–9.99/100,000 inhab.
		High: 2.50–4.99/100,000 inhab.
		Medium: 0.50–2.49/100,000 inhab.
		Low: <0.5/100,000 inhab.

Rate of new leprosy cases with grade 2 physical disability at the time of diagnosis/100,000 inhabitants.	Evaluates deformities caused by leprosy in the general population and compares them with other debilitating diseases.	The reduction trend of detection rate, followed by decreasing of this indicator, characterizes a reduction of the endemic disease's magnitude.

In the study period, 8238 new leprosy cases were identified, 6.25% (*n* = 515) in children under 15 and 7.27% (*n* = 599) in new leprosy cases with grade 2 disability. Of the 75 municipalities, only two had an increasing trend of the general detection coefficient: Carira (AAPC = 59.2%) and Moita Bonita (AAPC = 27.3%). Eight municipalities had a decreasing trend (Canindé do São Francisco, Cumbe, Estância, Ilha das Flores, Japoatã, Santa Luiza do Itanhy, Santana do São Francisco, and Santos Amaro das Brotas). In this group, the average annual reduction was 22.56% (Fig. 1).

**Figure 1:**
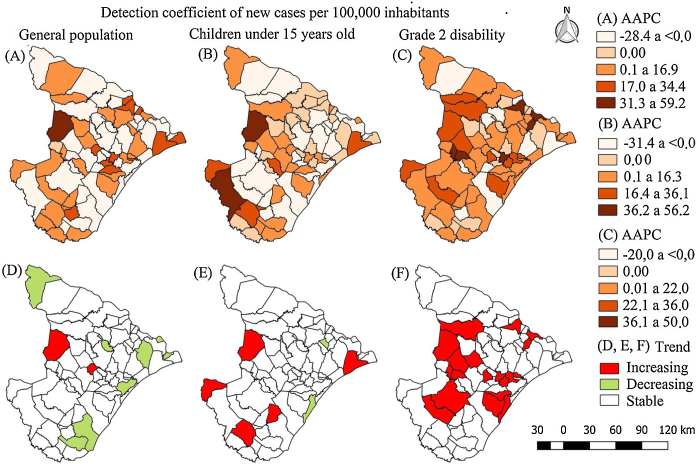
Spatial distribution of average annual percent changes and classification of the trend of leprosy magnitude indicators in Sergipe, Brazil, 2001–2015. AAPC, average annual percent change.

Regarding the detection coefficient in children under 15, five municipalities presented an increasing trend (Carira, Itabaianinha, Pacatuba, Poço Verde, and Salgado), with emphasis on Carira (AAPC = 56.2%). Only Aracaju (AAPC = −8.8%) and Malhada dos Bois (AAPC = −2.0%) had a decreasing trend for detection rate in children. For grade 2 disability, 19 municipalities had an increasing trend, especially Propriá (AAPC = 50.0%) and Divina Pastora (AAPC = 38.30%). No municipality presented any trend of reduction in the grade 2 disability indicator. This group had an average increasing trend of 29.3% (Fig. 1).

Although leprosy burden has declined over recent years,^2^, ^3^ recent studies have suggested that the number of patients registered in official information systems is substantially lower than the number of individuals with leprosy in Brazil.^6^ Studies performed in areas considered low endemicity have evidenced underdiagnosis and, therefore, a high hidden leprosy prevalence.^7^, ^8^

The highest number of municipalities with a trend of increase in the detection in children under 15 (*n* = 5) and the detection of new leprosy cases having grade 2 disability (*n* = 9) suggests the maintenance of leprosy transmission in Sergipe, a hidden leprosy prevalence, underdiagnosis of leprosy, and failures of the leprosy control programs in municipalities.^1^, ^3^, ^9^ The mismatch between the three indicators has already been evidenced in investigations carried out in the states of Bahia^9^ and Alagoas,^10^ which have geographical boundaries with Sergipe.

The detection coefficient of new leprosy cases with grade 2 disability is one of the most important indicators for evaluating the disease, and suggests a late diagnosis of leprosy.^3^ In Sergipe, municipalities with a tendency of increase of this indicator should receive special attention from the policymakers, especially through actions that facilitate early diagnosis.

Despite the advances observed in the reduction of general detection coefficient and in children under 15, leprosy still represents a public health problem in Sergipe.

## Financial support

None declared.

## Authors’ contributions

Carlos Dornels Freire de Souza: Statistical analysis; approval of final version of the manuscript; conception and planning of the study; drafting and editing of the manuscript; collection, analysis, and interpretation of data; participation in design of the study; intellectual participation in the propaedeutic and/or therapeutic conduct of the studied cases; critical review of the literature.

Thiago Cavalcanti Leal: Statistical analysis; approval of final version of the manuscript; conception and planning of the study; drafting and editing of the manuscript; collection, analysis, and interpretation of data; participation in design of the study; intellectual participation in the propaedeutic and/or therapeutic conduct of the studied cases; critical review of the literature; critical review of the manuscript.

João Paulo Silva de Paiva: Statistical analysis; approval of final version of the manuscript; conception and planning of the study; drafting and editing of the manuscript; collection, analysis, and interpretation of data; participation in design of the study; intellectual participation in the propaedeutic and/or therapeutic conduct of the studied cases; critical review of the literature; critical review of the manuscript.

Victor Santana Santos: Statistical analysis; approval of final version of the manuscript; conception and planning of the study; drafting and editing of the manuscript; collection, analysis, and interpretation of data; participation in design of the study; intellectual participation in the propaedeutic and/or therapeutic conduct of the studied cases; critical review of the literature; critical review of the manuscript.

## Conflicts of interest

None declared.
